# Supplementary material for: On-farm evaluation of growth and reproductive performances of Washera and Gumuz sheep in northwestern Ethiopia: Basics for setting up breeding objectives/goals
Source: PLoS One. 2021 Jul 20;16(7):e0254924. doi: 10.1371/journal.pone.0254924 (PMC8291677; doi:10.1371/journal.pone.0254924)
Supplement: Attachment — Submitted filename: PONE-D-21-11779_reviewer Sheep-Ethiopia.pdf [file pone.0254924.s003.pdf]

PLOS ONE

On-farm evaluation of growth and reproductive performances of Washera and Gumuz sheep in northwestern Ethiopia: basics for setting up breeding objectives/goals

--Manuscript Draft--

**Manuscript Number:**	PONE-D-21-11779
**Article Type:**	Research Article
**Full Title:**	On-farm evaluation of growth and reproductive performances of Washera and Gumuz sheep in northwestern Ethiopia: basics for setting up breeding objectives/goals
**Short Title:**	On-farm evaluation of growth and reproductive performances of Washera and Gumuz sheep
**Corresponding Author:**	Sisay Asmare<br>Debre Markos University,Burie campus<br>Burie, ETHIOPIA
**Keywords:**	ewe; growth and reproductive performance; lamb; productivity; traits
**Abstract:**	Growth and reproductive performance traits are traits of economic importance for sheep selection and productivity improvement interventions. This study aimed at comparative evaluation of growth and reproductive performance traits of sheep in the highland and lowland agro-ecologies of northwestern Ethiopia. Data on growth performance traits were collected from 144 Washera (78 male and 66 female) lambs and 72 Gumuz (37 male and 35 female) lambs. Data on reproductive performance traits were collected from 260 Washera (130 rams and 130 ewes) sheep and 150 Gumuz (75 rams and 75 ewes) sheep. General linear model univariate procedure was employed to analyze the collected data. Mean±SD values of adjusted birth weight were 2.16±0.54 and1.62±0.55 for Washera and Gumuz sheep, respectively. Mean±SD values of adjusted weaning weight were 8.59±0.79 and 7.44±1.26 kg for Washera and Gumuz sheep, respectively. Mean±SD values of adjusted yearling weight of Washera and Gumuz sheep was 21.80±1.91 and 20.37±1.85 kg, respectively. Pre-weaning average daily weight gain for Washera was 70 g/day much better performance than 60 g/day of Gumuz. Breed type affected live weight significantly (P<0.001) at all age groups. Mean±SD values of age at first lambing, lambing interval and annual reproductive rate of Washera sheep were 11.69±1.96 months, 9.27±3.53 months and 2.12 lambs, respectively. The corresponding values for Gumuz sheep were 12.51±1.95 months, 10.43±1.25 months and 1.71 lambs, respectively. Production and reproduction performance values for traits of Washera and Gumuz sheep were comparable to other Ethiopian indigenous breeds and African breeds as well. Production and reproduction performance values of traits varied across the two breeds and sexes as well. These values can be used to set up breeding objectives or goals for selective breeding of sheep by giving special emphasis to growth traits, believed to have medium heritability values.
**Order of Authors:**	Sisay Asmare
	Kefyalew Alemayehu
	Solomon Abegaz K
	Aynalem Haile
**Additional Information:**	
**Question**	**Response**
**Financial Disclosure**<br><br>Enter a financial disclosure statement that describes the sources of funding for the work included in this submission. Review the submission guidelines for detailed requirements. View published research	Sisay Asmare received fund from Biotechnology Research Institute of Bahir Dar University and Ministry of Education via College of Agriculture and Environmental Sciences of Bahir Dar University ordered by a letter written with a Reference number of 1/8048/14-10 and 1/2432/13 4, respectively. The website for College of Agriculture and Environmental Sciences of Bahir Dar University is https://bdu.edu.et/caes/. The funders do not take part or had no role in study conceptualization and design, data collection and analysis, decision to publish, or preparation of the manuscript.

Powered by Editorial Manager® and ProduXion Manager® from Aries Systems Corporation

articles from *PLOS ONE* for specific examples.<br><br>This statement is required for submission and **will appear in the published article** if the submission is accepted. Please make sure it is accurate.<br><br>**Unfunded studies**<br>Enter: *The author(s) received no specific funding for this work.*<br><br>**Funded studies**<br>Enter a statement with the following details:<br>• Initials of the authors who received each award<br>• Grant numbers awarded to each author<br>• The full name of each funder<br>• URL of each funder website<br>• Did the sponsors or funders play any role in the study design, data collection and analysis, decision to publish, or preparation of the manuscript?<br>• **NO** - Include this sentence at the end of your statement: *The funders had no role in study design, data collection and analysis, decision to publish, or preparation of the manuscript.*<br>• **YES** - Specify the role(s) played.<br><br>* typeset	
**Competing Interests**<br><br>Use the instructions below to enter a competing interest statement for this submission. On behalf of all authors, disclose any competing interests that could be perceived to bias this work—acknowledging all financial support and any other relevant financial or non-financial competing interests.<br><br>This statement is required for submission and **will appear in the published article** if the submission is accepted. Please make sure it is accurate and that any funding sources listed in your Funding Information later in the submission form are also declared in your Financial Disclosure statement.	The authors have declared that no competing interests exist

View published research articles from *PLOS ONE* for specific examples.<br><br>**NO authors have competing interests**<br><br>Enter: *The authors have declared that no competing interests exist.*<br><br>**Authors with competing interests**<br><br>Enter competing interest details beginning with this statement:<br><br>*I have read the journal's policy and the authors of this manuscript have the following competing interests: [insert competing interests here]*<br><br>* typeset	
**Ethics Statement**<br><br>Enter an ethics statement for this submission. This statement is required if the study involved:<br><br>• Human participants<br>• Human specimens or tissue<br>• Vertebrate animals or cephalopods<br>• Vertebrate embryos or tissues<br>• Field research<br><br>Write "N/A" if the submission does not require an ethics statement.<br><br>General guidance is provided below. Consult the submission guidelines for detailed instructions. **Make sure that all information entered here is included in the Methods section of the manuscript.**	Earlier to the study, data collection formats and procedures were reviewed and agreed by the research ethics review committee of Debre Markos University, Ethiopia (Ref. number DMU 10/2018 E.C). In addition, target group smallholder farmers provided their verbal informed consent to participate in this study. Besides, the data were analyzed namelessly; ethnicity and religious issues were not asked and recorded during data collection.

Powered by Editorial Manager® and ProduXion Manager® from Aries Systems Corporation

**Format for specific study types**<br><br>**Human Subject Research (involving human participants and/or tissue)**<br>• Give the name of the institutional review board or ethics committee that approved the study<br>• Include the approval number and/or a statement indicating approval of this research<br>• Indicate the form of consent obtained (written/oral) or the reason that consent was not obtained (e.g. the data were analyzed anonymously)<br><br>**Animal Research (involving vertebrate animals, embryos or tissues)**<br>• Provide the name of the Institutional Animal Care and Use Committee (IACUC) or other relevant ethics board that reviewed the study protocol, and indicate whether they approved this research or granted a formal waiver of ethical approval<br>• Include an approval number if one was obtained<br>• If the study involved *non-human primates*, add *additional details* about animal welfare and steps taken to ameliorate suffering<br>• If anesthesia, euthanasia, or any kind of animal sacrifice is part of the study, include briefly which substances and/or methods were applied<br><br>**Field Research**<br><br>Include the following details if this study involves the collection of plant, animal, or other materials from a natural setting:<br>• Field permit number<br>• Name of the institution or relevant body that granted permission	
**Data Availability**<br><br>Authors are required to make all data underlying the findings described fully available, without restriction, and from the time of publication. PLOS allows rare exceptions to address legal and ethical concerns. See the PLOS Data Policy and FAQ for detailed information.	Yes - all data are fully available without restriction

A Data Availability Statement describing where the data can be found is required at submission. Your answers to this question constitute the Data Availability Statement and **will be published in the article**, if accepted.<br><br>**Important:** Stating 'data available on request from the author' is not sufficient. If your data are only available upon request, select 'No' for the first question and explain your exceptional situation in the text box.<br><br>Do the authors confirm that all data underlying the findings described in their manuscript are fully available without restriction?	
**Describe where the data may be found in full sentences. If you are copying our sample text, replace any instances of XXX with the appropriate details.**<br><br>• If the data are **held or will be held in a public repository**, include URLs, accession numbers or DOIs. If this information will only be available after acceptance, indicate this by ticking the box below. For example: *All XXX files are available from the XXX database (accession number(s) XXX, XXX.).*<br>• If the data are all contained **within the manuscript and/or Supporting Information files**, enter the following: *All relevant data are within the manuscript and its Supporting Information files.*<br>• If neither of these applies but you are able to provide **details of access elsewhere**, with or without limitations, please do so. For example:<br><br>*Data cannot be shared publicly because of [XXX]. Data are available from the XXX Institutional Data Access / Ethics Committee (contact via XXX) for researchers who meet the criteria for access to confidential data.*<br><br>*The data underlying the results presented in the study are available from (include the name of the third party*	All relevant data are within the manuscript and its Supporting Information files

and contact information or URL).<br>• This text is appropriate if the data are owned by a third party and authors do not have permission to share the data.<br><br>* typeset	
Additional data availability information:	Tick here if the URLs/accession numbers/DOIs will be available only after acceptance of the manuscript for publication so that we can ensure their inclusion before publication.

Powered by Editorial Manager® and ProduXion Manager® from Aries Systems Corporation

Manuscript Click here to access/download;Manuscript;manuscript.doc

On-farm evaluation of growth and reproductive performances of Washera and Gumuz sheep in northwestern Ethiopia: basics for setting up breeding objectives/goals

Sisay Asmare[1*¶], Kefyalew Alemayehu[2&], Solomon Abegaz k.[3&], Aynalem Haile[4&]

[1]Debre Markos University, Burie campus, P.o.Box 18, Ethiopia

[2]Bahir Dar University, College of Agriculture and Environmental Sciences, Department of Animal Science and Technology, Bahir Dar, Ethiopia

Email: - bdubiotechnology@gmal.com

[3] Ethiopian Institute of Agricultural Research, Addis Ababa, Ethiopia

Email: - solo.abegaz@gmail.com

[4]International Center for Agricultural Research in the Dry Areas (ICARDA), Addis Ababa, Ethiopia

Email: - a.haile@cgiar.org

*Corresponding Author: Email:- sisayasmbel@yahoo.com

: ORCID:- 0000-0003-2332-2937

Abstract

Growth and reproductive performance traits are traits of economic importance for sheep selection and productivity improvement interventions. This study aimed at comparative evaluation of growth and reproductive performance traits of sheep in the highland and lowland agro-ecologies of northwestern Ethiopia. Data on growth performance traits were collected from 144 Washera (78 male and 66 female) lambs and 72 Gumuz (37 male and 35 female) lambs. Data on reproductive performance traits were collected from 260 Washera (130 rams and 130 ewes) sheep and 150 Gumuz (75 rams and 75 ewes) sheep.

General linear model univariate procedure was employed to analyze the collected data. Mean±SD values of adjusted birth weight were 2.16±0.54 and [illegible]2±0.55 for Washera and Gumuz sheep, respectively. Mean±SD values of adjusted weaning weight were 8.59±0.79 and 7.44±1.26 kg for Washera and Gumuz sheep, respectively. Mean±SD values of adjusted yearling weight of Washera and Gumuz sheep was 21.80±1.91 and 20.37±1.85 kg, respectively. Pre-weaning average daily weight gain for Washera was 70 g/day much better performance than 60 g/day of Gumuz. Breed type affected live weight significantly (P<0.001) at all age groups. Mean±SD values of age at first lambing, lambing interval and annual reproductive rate of Washera sheep were 11.69±1.96 months, 9.27±3.53 months and 2.12 lambs, respectively. The corresponding values for Gumuz sheep were 12.51±1.95 months, 10.43±1.25 months and 1.71 lambs, respectively. Production and reproduction performance values for traits of Washera and Gumuz sheep were comparable to other Ethiopian indigenous breeds and African breeds as well. Production and reproduction performance values of traits varied across the two breeds and sexes as well. These values can be used to set up breeding objectives or goals for selective breeding of sheep by giving special emphasis to growth traits, believed to have medium heritability values.

Key words: ewe; growth and reproductive performance; lamb; productivity; traits

Introduction

Since domestication, sheep have become essential parts of farms across the world [1] and comprises 16 percent of the total TLUs in sub-Saharan Africa [2]. In part it is because of their ability to adapt to local environments [1]. Small ruminants represent only 7% of the average total capital invested in livestock in the mixed crop-livestock production system, but they account on average for 40% of the cash income and 19% of the total value of subsistence food derived from all livestock production [3-4]. Sheep and goats contribute a quarter of the domestic meat consumption; about half of the domestic wool requirements; about 40% of fresh skins and 92% of the value of semi-processed skin and hide export trade. The increased domestic and international demand for Ethiopian sheep and goats has established them as important sources of Inland Revenue as well as foreign currency [3].

Therefore, integrated attempt with emphasis on prescribing and genetic enhancement is crucial for improving animal output by improving growth and fertility traits [5]. In Ethiopia, there are 32.85 million sheep, more than 99% of which are indigenous [6]. However, the production of local sheep under traditional production system is low with high mortality of lambs [7]. Because of this the increasing need for food of animal origin has largely been met with increasing number of sheep while productivity per sheep has remained low [8]. Live weight and growth rate are economically critical features, requiring particular attention in any breeding program intended to improve overall productivity since lambs are mainly raised for mutton [7]. Besides, good reproductive performance is a prerequisite for any successful genetic improvement and it determines production efficiency which depends on various factors including age at first lambing, litter size, lambing interval and the life time productivity of the ewe [9-11]. Indigenous sheep breeds are compatible genotypes for the low input traditional production system since, indigenous sheep breeds have special adaptive features such as: tolerance to a wide range of diseases, water scarcity tolerance and ability to better utilize the limited and poor quality feed resources [12]. Indubitably, conservation and utilization of these local breeds of sheep is part of animal husbandry and should, ideally, be based on complete information on distribution, structures, and trends, productive and adaptive performances of populations of the existing breeds [13].

Washera and Gumuz indigenous sheep were produced under highland cereal-livestock and lowland crop-livestock sheep production systems, respectively [14]. Morphologically, Washera is a short fat tail, large body size, short-haired, predominantly brown, both males and females are polled reared by Amhara and Agew communities. Washera is a good meat producer under good environment [15]. Gumuz sheep is long thin tail, somewhat dwarf, convex face profile, long pendulous ear, commonly plain brown or with patch coat color, polled and reared by Gumuz and Amhara communities [14]. Gumuz sheep is adapted to heat and have unique genetic make-up [15]. On-farm monitoring involves monitoring the productive and reproductive performance of a breed on selected representative village flocks or herds [16]. Previous study has been conducted on growth performance of Washera sheep in Quarit and Yelmandensa districts of Amhara region, Ethiopia [17]. Nevertheless, most Ethiopian sheep breed types are characterized by high within-population variability, have undergone little selection for improved meat production and true breed potentials are not known [18, 19].

In Ethiopia , researches regarding on - farm performance of indigenous sheep and their crosses with exotic breeds are not well grounded due to the reason that many researchers has tended to focus on-station performance evaluation rather than on farm condition [20]. Yet, on-farm performance evaluation gives a more representative performance level of the breed since it is undertaken under the natural production environment of the breed [16]. Thus, the objective of this study was to evaluate growth and reproductive performance of Washera and Gumuz indigenous sheep and generate information for further establishment of selective breeding and productivity improvement programs under low input sheep production systems of highlands and lowlands of northwestern Ethiopia. We hypothesized that the performance values of growth and reproductive traits of Washera and Gumuz sheep populations was not comparable to other Ethiopian and African breeds and there are no variations in performance values of growth and reproductive traits between Washera and Gumuz sheep populations of northwestern Ethiopia.

Materials and Methods

Ethics statement

Earlier to the study, data collection formats and procedures were reviewed and agreed by the research ethics review committee of Debre Markos University, Ethiopia (Ref. number DMU 10/2018 E.C). In addition, target group smallholder farmers provided their verbal informed consent to participate in this study. Besides, the data were analyzed namelessly; ethnicity and religious issues were not asked and recorded during data collection.

Geographic coordinates of the study areas

This study was conducted in two districts, Burie and Mandura considered as potential areas for Washera and Gumuz sheep breed types in North Western Ethiopia (Fig 1). According to altitudinal location, rainfall and crop yields, the agro-ecological zone of Burie is highland and the agro-ecological zone of Mandura is lowland [21].

Fig 1. Map of the study districts

Sampling size and sampling methods

After preliminary survey, two districts Burie and Mandura were selected by purposive sampling method [22] based on sheep production potential. Similarly, two rural peasant associations (Wanegedam and Daez Baguna) were selected from their respective districts. The key informants including the development agents and peasant association administrators were given training to enumerate smallholders engaged in sheep production keeping three sheep or more and how to fill questionnaires.

In the mean time, from a total of 1103 households of Burie district engaged in sheep production, 260 households owning ram and ewe giving birth at commencement of data collection were selected by random sampling method. A total of 260 Washera (130 ram and 130 ewes) sheep were considered for the monitoring of reproductive performance. A total of 144 Washera (78 male and 66 female) lambs were considered for the monitoring of growth performance.

Similarly, from a total of 622 households of Mandura district engaged in sheep production, 150 households owning ewe giving birth at commencement of data collection were selected by probability random sampling method [22]. A total of 150 Gumuz (75 rams and 75 ewes) sheep were considered for the monitoring of reproductive performance. A total of 72 Gumuz (37 males and 35 females) lambs were considered for the monitoring of growth performance.

Data collection and animal management

The necessary data on growth and reproductive parameters were collected from sheep flocks managed under mixed - crop livestock farming system of Burie and Mandura districts. Data collection was carried out for two years from September, 2017 to October, 2018 G.C. Smallholders owning rams, ewes and lambs were asked for voluntarily for sparing their sheep and time while recording monitoring data on growth and reproductive performance traits. Live weight measurements were taken five times at the age of day 1, 90 days, 180 days, 270 days and 365 days. Live weight measurements were taken in the mornings, before the

animals left the shelter to graze to avoid undesirable variations because of changes in rumen volumes [23, 24]. Lamb weight measurements were taken taking ethical and animal welfare into account. Suspended weighing scale, rope, tripod stick and soil fertilizer bags were used for measuring live weight of lambs. Concurrently, date of birth, birth weight, type of birth, sex of lamb, season of birth, lambing interval, age at first lambing, age at first mating, number of lambs born during life time of the ewe, reproductive age of the ewe, number of offspring produced, number of offspring weaned and ewe parity were recorded starting from 24 hours of the new born by the recruited enumerators in each respective study site. During the course of data collection, dams and lambs were managed to graze in communal grazing lands and crop aftermaths for an average of eight hour and supplemented with crop residues and *atella* (bi-product of local brewery). Different formulas were used to calculate some parameters:

Weight records at various ages were adjusted for actual age using different formulas used by [25, 26].

$$\text{Adjusted Weaning Wt (kg)} = 90(W2-W1)/D + W1 \quad (1)$$

$$\text{Adjusted 6 Month Wt (kg)} = 180(W3-W1)/D + W1 \quad (2)$$

$$\text{Adjusted Yearling Wt (kg)} = 365(W4-W1)/D + W1 \quad (3)$$

Where, W2, W3 and W4 = weight at a given age

W1 = birth weight

Wt=weight

D = number of days between weighing date and date of birth

And average daily weight gain was calculated by the formulas:

Average daily weight gain from birth to weaning (g)

$$= (AWWT(kg) - BWT(kg))/90 * 1000 \quad (4)$$

Average daily weight gain from weaning to 6 month age (g)

$$= (A6MWT(kg) - AWWT(kg))/90 * 1000 \quad (5)$$

Average weight gain from 6 month age to 365 days yearling (g)

$$= (AYWT9(kg) - AWWT(kg))/275 * 1000 \quad (6)$$

Where :

BWT = Birth weight

AWWT = Adjusted weaning weight at 90 days

A6MWT = Adjusted 6 month weight at 180 days

AYWT = Adjusted yearling weight at 365 days

Annual reproductive rate (ARR) of Washera and Gumuz ewes was calculated by a formulae adopted from [27] :

Annual reproductive rate (ARR)

= 365 * average litter size/average days of lambing interval…….(7)

Lamb survival rate was calculated by the formulae adopted from [28]:

Lamb survival rate (%)

= (Number of offspring weaned/ number of offspring produced) *100 ………(8)

Statistical analysis

Data on birth weight, weaning weight, six month and yearling weight at different age groups were collected and later converted to birth weight, adjusted weaning weight , adjusted 6 month weight , adjusted yearling weight, pre weaning and post weaning average daily weight gain . The data collected on growth and reproductive performance traits were arranged in an excel spread sheet. Ultimately, the data were analyzed using General Linear Model(GLM) univariate procedure of Statistical Package for Social Science for window version 20.0, SPSS (2011) [29] fitting growth rate traits (birth weight, adjusted weaning weight, adjusted 6 month weight, adjusted yearling weight, pre weaning and post weaning average daily weight gain) and reproductive performance traits such as age at first lambing, lambing interval, annual reproductive rate, number of lambs born per ewe life time as dependent variable and non-genetic factors such as breed, sex of lamb, parity of the dam, season of birth and type of birth as fixed factor(s). In the mean time mean±SD and level of significance from tests of between subject effects were summarized into tabular form. Non significant effects of independent variable (non-genetic factors) were not reported. Data

analysis of growth and reproductive performance traits were carried out using two different statistical models:

The statistical model for analyzing growth performance was written as follows:

$Y_{ijklmno} = \mu + B_i + S_j + P_k + S_l + T_m + (B_i x S_l)_n + (B_i x T_m)_o + e_{ijklmno}$ …….. ….. (mode1 1)

Where :

$Y_{ijklmno}$ = the body weight and average daily weight gain of the *n*th lamb of nth growing lamb

μ=population mean

B_i= effect of ith breed (Washera, Gumez)

S_j = effect of jth sex of lamb (male or female)

P_k= effect of kth parity of the dam (P=first, second, third and fourth)

S_l= effect of lth season of birth (B(*belg*)= short rainy season (March – May), S=cold dry season (September-November), DS = Dry Season (December-February), RS=Rainy Season (June-August)

T_m= effect of mth type of birth (single, twin)

$B_i x S_l$= interaction effect of ith breed and lth season of birth

$B_i x T_m$= interaction effect of ith breed and mth type of birth

$E_{ijklmno}$= error/residual effect

The statistical model for analyzing reproductive performance was written as follows:

$Y_{ijklm} = \mu + B_i + S_j + P_k + T_l + (PxT)_m + e_{ijklm}$…………….. (model 2)

Where:

Yijklmn= reproductive performance traits (age at first lambing, lambing interval, annual reproductive rate, number of lambs born per ewe life time)

U –population mean

B_i – the effect of ith breed (Washera, Gumuz)

S_j = the effect of jth sex of lamb (male, female)

P_k= the effect of kth parity of the dam (P=first, second, third, fourth)

T_l= the effect of mth type of birth (single, twin)

$(P_k x T_l)_m$= the interaction effect of nth parity and type of birth

E_{ijklm}= Error/residual effect

Results

Growth performance of Washera and Gumuz sheep

The result of this study indicated that there exists variation in growth rate between Washera and Gumuz sheep populations. Table 1 depicts live weight and factors affecting live weight at different age groups. Mean±SD values of adjusted birth weight were 2.16±0.54 and 1[illegible]62±0.55 for Washera and Gumuz sheep, respectively. Mean±SD values of adjusted weaning weight were 8.59±0.79 and 7.44±1.26 kg for Washera and Gumuz sheep, respectively. Pre-weaning average daily weight gain for Washera was 70 g/day much better performance than 60 g/day of Gumuz. The minimum and maximum values of live weight for Washera and Gumuz sheep were shown in Fig 2. For instance, the minimum weaning weight of Gumuz sheep is 5.03 kg and the maximum value is 10.09 kg. The minimum and maximum weights of Washera sheep at six months of age were 10.89 and 17.83 kg, respectively. The minimum and maximum weights of Gumuz sheep at six months of age were 8.91 and 16.84 kg, respectively. The minimum and maximum yearling weights of Washera sheep were 19.04 and 25.06 kg, respectively. The minimum and maximum yearling weights of Gumuz sheep were 16.04 and 24.06 kg, respectively.

Table 1. Mean± SD values of live weight and tests of between subject effects

Factors	N	BWT	AWWT	A6MWT	AYWT
		Mean±SD	Mean± SD	Mean± SD	Mean± SD
Breed		***	***	***	***
Overall	216	1.98±0.60	8.22±0.11	13.35±1.16	21.33±2.00
Washera	144	2.16±0.54	8.59±0.79	13.94±1.2	21.8±1.91
Gumuz	72	1.62±0.55	7.45±1.26	12.16±1.67	20.37±1.85
Sex		***	ns	*	*
Overall	216	1.98±0.60	8.22±1.11	13.35±1.61	21.33±2.00
Male	115	2.19±0.52	8.41±1.09	13.87±1.47	22.08±1.78
Female	101	1.73±0.59	7.99±1.09	12.75±1.56	20.47±1.90
Parity		ns	ns	ns	***
Overall	216	1.98±0.60	8.22±1.11	13.35±1.61	21.33±2.00
1	74	1.78±0.66	7.75±1.14	12.34±1.46	19.67±1.12
2	67	2.15±0.54	8.63±0.79	14.24±1.23	22.9±1.33
3	40	2.08±0.53	8.49±1.13	14.09±1.24	22.98±1.35
4	35	1.94±0.53	8.08±1.16	12.9±1.56	19.9±1.03
Season of birth		ns	*	**	***
Overall	216	1.98±0.60	8.22±1.11	13.35±1.61	21.33±2.00
Short rainy season(March – May)	54	2.12±0.53	8.88±0.63	14.07±0.86	21.68±0.93
Rainy Season(June-August)	74	2.08±0.57	8.42±1.10	14.23±1.37	23.36±1.13
Cold dry season(September-November)	32	1.85±0.57	7.75±1.14	12.6±1.62	20.44±0.79
Dry Season (December-February)	65	1.83±0.65	7.77±1.08	12.26±1.43	19.3±1.34
Type of birth		***	*	**	ns
Overall	216	1.98±0.60	8.21±1.11	13.35±1.61	21.74±2.00
Single	92	2.48±0.60	8.57±1.15	13.95±1.49	21.74±2.00
Twin	124	1.6±0.60	7.95±1.08	12.89±1.70	21.02±2.00
B_ixS_l	216	***	***	***	***
B_ixT_m	216	***	**	***	***

SD = standard deviation, BWT =birth weight, AWWT = adjusted weaning weight, A6MWT = adjusted six month weight, AYWT =adjusted yearling weight, Bi=breed, SL=season of birth, Tm=type of birth, values, B_ixS_l= the interaction effect between breed and season of birth , B_ixT_m= the interaction effect between breed and type of birth, * =significant at $p<0.05$, **=significant at $p<0.01$, ***= very high significant at $p<0.001$, ns = non significant

Fig 2. Minimum and maximum values of live weight in kg at different age groups

Reproductive performance of Washera and Gumuz sheep

Measures of reproduction commonly used in sheep and goats include age at puberty, age at first lambing/kidding, post-partum interval, and parturition interval and fertility indices [10]. Average age of first mating for males, average age of first mating for females, average age at first lambing of ewes, average lambing interval of ewes, litter size, annual reproductive rate, average reproductive age of ewes and number of lambs born during life time of the ewe of Washera sheep was 6.18±1.85 months, 6.52±1.30 months, 11.69±1.96 months, 9.27±3.53 months, 1.62 lambs/birth, 2.12 lambs/ewe/year (author calculation), 6.63±1.87 years and 12.27±4.56 lambs, respectively.

Similarly, average age of first mating for males, average age of first mating for females, average age at first lambing of ewes, average lambing interval of ewes, litter size, annual reproductive rate, average reproductive age of ewes and number of lambs born during life time of the ewe of Gumuz sheep was 7.27±2.00 months, 7.64±1.67 months, 12.51±1.95 months, 10.43±1.25 months, 1.43±0.49 lambs/birth, 1.71 lambs/ewe/year (author calculation), 6.67±0.17 years and 9.89 lambs, respectively.

The impact of reproduction on sheep and goat productivity is best estimated by the annual reproductive rate [9]. Long-term studies carried out over periods of several years have shown that the annual reproductive rate (number of young produced per breeding female per year) varies from 1.5 to over two lambs [27]. This rate of reproduction results in part is from the uncontrolled access of rams to ewes on a permanent basis and in part from the litter size ; in that annual reproductive rate(ARR) is a function of litter size and parturition interval(ARR=Liter size*365/ parturition interval. ARR of this study was calculated by a formulae adopted from [27].

ARR for Washera ewe is

= (365 * average litter size)/average days of lambing interval

ARR for Gumuz ewe is

= (365 * average litter size)/average days of lambing interval

Hence; ARR was 2.12 and 1.71 lambs/ewe/year for Washera and Gumuz sheep, respectively. Annual reproductive rate of Washera (2.12 lambs/ewe/year) and Gumuz (1.71 lambs/ewe/year) was within the range of [27].

Lamb survival rate was calculated by the formulae used by [28]:

Lamb survival rate (%)

= (number of offspring weaned/number of offspring produced)*100

Thus, lamb survival rate were 70.7 % and 70.1 % for Washera and Gumuz sheep, respectively. Lambing interval of Washera ewe is 8 to 24 months. Lambing interval of Gumuz ewe is 6 to 12 months. Number of lambs born per ewe life time of Washera sheep is 3(minimum) and 21 (maximum). Number of lambs born per ewe life time of Gumuz sheep is 4 (minimum) and 16 (maximum).

Discussion

Growth performance and the effect of factors

Mean±SD values of birth weight of Washera lamb was close to 2.2±0.04 kg of Wollo highland lambs [30] and 2.4±0.2 kg of Bonga sheep [31] under village management condition. Yearling weight values of Washera and Gumuz sheep were close to 23.7 ± 0.04 kg of Horro sheep [32].

Growth and reproductive performance of indigenous sheep could be improved through improvement of genetics and environment. However, growth and reproductive performance of indigenous sheep was influenced by most of the non-genetic factors [35, 38] and it is thus appropriate to consider the factors affecting their outcome. The current study revealed similar result. For instance, breed , the interaction effect between breed and season of birth and the

interaction effect between breed and type of birth all exerted significant effec[illegible]<0.01) on live weight at all age groups. Sex affected live weight at all age groups significantly (P<0.05) except on weaning weight. Type of birth affects birth weight significantly (P<0.05). Larger birth weight was recorded for single born lambs than their twin contemporaries. This is due to the fact that single born lambs have got a better chance of getting good nourishment at the fetal stage. The effects of breed, sex and type of birth on birth weight were in agreement with [7] for Horro and Menz sheep. Season of birth affects weaning weight significantly (P<0.05). Lambs born during rainy season (June-August) and short rainy season (March – May) of the year were found to be heavier than lambs born during cold dry season (September-November) and dry season (December-February). This could be due to feed availability during these seasons for the dam to meet its production requirement for lactation beyond maintenance requirement to suckle the lamb until weaning and to the lamb side to graze on green pasture from weaning onwards. Parity and season of birth were non-genetic factors exerting significant (P<0.001) effect on yearling weight. Larger yearling weight observed during 2nd and 3rd parities. Season of birth affects yearling weight significantly (P<0.001). Lambs born during rainy season (June-August) and short rainy season (March – May) of the year were found to be heavier than lambs born during cold dry season (September-November) and dry season (December-February). This could be due to feed availability during these seasons for the lamb to graze on green pasture. Breed type affected pre-weaning average daily weight gain significantly (P<0.01). The variation in pre-weaning average daily weight gain between Washera and Gumuz sheep concur with [19], suggesting that wide variability exists among Ethiopian small ruminant breeds with respect to potential growth rates and mature weight. Type of birth affected pre-weaning average daily weight gain significantly (P<0.01). This result was in consistence with [7] report for Horro and Menz sheep. The post-weaning growth rate of Washera sheep was comparable and even better than some other indigenous breeds indicating its potential for meat (mutton) production for the local and export markets [33]. For instance, post-weaning growth rate of Washera sheep found by this study was 60 g/day, which was close to 63.4±4.0 of Bonga sheep [32].

Reproductive performance and the effect of factors

The overall mean±SD value for average age of first mating for females was 6.93±1.54 months (207.9 days). These ewes were early maturing than Djallonké gimmers of Ghana mated when they are 8 months (240 days) old [34]. Litter size of Washera (1.62 lambs/birth) and Gumuz (1.43±0.49 lambs/birth) sheep was higher than 1.34 ± 0.01 of Horro sheep [33] in South Western Ethiopia. The larger litter size of Washera and Gumuz sheep indicates that these sheep populations were prolific compared to other Ethiopian indigenous sheep and most African breeds. Lambing interval of Washera ewe is 8 to 24 months. Lambing interval of Gumuz ewe is 7 to 12 months. The overall mean±SD value for lambing interval of this study was 9.69±2.96 months (290.7 days). This lambing interval was longer than 262 ±53.4 days of sheep in South Western part of Ethiopia [35]. However, improving the management practice will make shorter parturition intervals (below 9.69±2.96 months) enabling approximately three lamb crops to be obtained in two years. ARR of Washera ewe (2.12 lambs/ewe/year) was found to be greater than 1.88 ± 0.44 of sheep in South Western part of Ethiopia [35]. Annual reproductive rate (ARR) of Gumuz ewe (1.71lambs/ewe/year) looks closer to 1.88 ± 0.44 of sheep in South Western part of Ethiopia [35]. ARR of Washera ewe (2.12 lambs/ewe/year) and of Gumuz ewe (1.71 lambs/ewe/year) under smallholder mixed farming system of North Western Ethiopia found to be better performance compared to 1.20 and 1.05 [27] for pastoral and agro-pastoral areas of Ethiopia, respectively. Minimum and maximum number of lambs born per ewe life time of Washera sheep is 3 and 4, respectively. Minimum and maximum number of lambs born per ewe life time of Gumuz sheep is 4 and 16, respectively. The overall mean±SD value for number of lambs born per ewe life time (NLBE) of Washera and Gumuz sheep was 11.4±4.13 lambs/ewe life time. NLBE of Washera and Gumuz sheep was greater than 9.31 lambs/ewe life time of sheep under mixed - crop livestock and 12.06 lambs/ ewe life time under pastoral sheep production systems of Ethiopia [36]. Lamb survival rate were 70.7 % and 70.1 % for Washera and Gumuz sheep, respectively. This comparable value of lamb survival rate between Washera and Gumuz sheep was attributed to similar village traditional type of sheep management practices under similar mixed crop livestock sheep production system to which these indigenous breeds are kept. Lamb survival rate of Washera and Gumuz were higher than 68 % of Horro sheep, but less than 83 % of Menz sheep [37]. Thus, the tendency of pre weaning lamb loss was higher for Washera and Gumuz than Menz sheep. This variation in lamb survival rate between Washera, Gumuz and Menz could be attributed to sheep management system within which these indigenous breeds were kept (on-farm Vs on-station). Moreover, the variation could be

explained by varied sheep production systems (mixed crop - livestock of Washera and Gumuz Versus sheep barley system of Menz sheep).

Tests between subject effects indicated that breed type exerts significant (P<0.05) effect on age at first lambing, lambing interval, annual reproductive rate and number of lambs born per ewe life time. However, the effect of breed type on litter size is non significant. Likewise, parity, birth type, sex of lamb and the interaction effect between parity and type of birth does not affect age at first lambing, lambing interval, annual reproductive rate and number of lambs born per ewe life time. Age at first lambing, lambing interval, annual reproductive rate and number of lambs born per ewe life time all vary across the two breed types (districts). This result was in agreement with the report for Bonga sheep [31] in Southern Ethiopia. But, this result was against to [35, 38] report, suggesting that, district has no influence on age at first lambing, lambing interval, annual reproductive rate and number of lambs born per life time of ewe traditionally managed in the South Western part of Ethiopia and Begayt sheep of North-Western Zone of Tigray Region, in Humera district, Northern part of Ethiopia.

Conclusion

This study was aimed at evaluating growth and reproductive performance traits of indigenous sheep breeds under on-farm sheep management conditions of northwestern Ethiopia. Analysis of the monitoring data revealed that mean±SD values of birth weight were 2.16±0.54 and1.62±0.55 for Washera and Gumuz sheep, respectively. Mean±SD values of weaning weight were 8.59±0.79 and 7.44±1.26 kg for Washera and Gumuz sheep, respectively. Pre-weaning average daily weight gain for Washera was 70 g/day much better performance than 60 g/day of Gumuz. Breed , the interaction effect between breed and season of birth and the interaction effect between breed and type of birth all exerted significant effect (P<0.01) on live weight at all age groups. Annual reproductive rate (ARR) was 2.12 and 1.71 lambs/ewe/year for Washera and Gumuz sheep, respectively. Lamb survival rate were 70.7 % and 70.1 % for Washera and Gumuz sheep, respectively. Production and reproduction performance values for traits of Washera and Gumuz sheep were comparable or greater than to other Ethiopian and African breeds/ecotypes. The variations between minimum and maximum values of growth and reproductive performance traits indicated the presence of within breed variation. Tests between subject effects indicated

between breed variation in values of growth and reproductive performance traits. Thus, the null hypothesis of the study has been rejected. The observed variations in values of growth and reproductive performance were essential steps for setting up breeding objectives or goals of sheep breeding program for selection within and among indigenous sheep populations in the highlands and lowlands of northwestern Ethiopia by giving special consideration to growth rate traits believed to have medium heritability values. In the mean time these values could be used as a valuable inputs for selective breeding and productivity improvement.

Acknowledgment

The authors sincerely thank smallholder farmers of the study areas for participating in restraining and handling of their sheep while measuring morphometric traits. We also wish to thank development agents of respective district agricultural offices for their role in data collection.

Funding Statement

Sisay Asmare received fund from Biotechnology Research Institute of Bahir Dar University and Ministry of Education via College of Agriculture and Environmental Sciences of Bahir Dar University ordered by a letter written with a Reference number of 1/8048/14-10 and 1/2432/13 4, respectively. The website for College of Agriculture and Environmental Sciences of Bahir Dar University is https://bdu.edu.et/caes/. The funders do not take part or had no role in study conceptualization and design, data collection and analysis, decision to publish, or preparation of the manuscript.

Competing interests

We authors declare that we do not have conflict of interest

Availability of data and material

The datasets used and/or analyzed during the current study available from the corresponding author on request.

Authors' contributions

All authors contributed to the study conception and design. Material preparation, data collection and analysis were performed by Sisay Asmare. The first draft of the manuscript was written by Sisay Asmare. Kefyalew Alemayehu, Solomon Abegaz K. and Aynalem Haile supervised the study and commented on previous versions of the manuscript. All authors read and approved the final manuscript.

References

1. Hirst KK. How and when sheep (Ovis Aries) were first domesticated; 2017. Available from: https://www.thoughtco.com/when-sheep-were-first-domesticated-172635

2. FAO (Food and Agricultural Organization). Cattle and small ruminant production systems in sub-Saharan Africa. A systematic review Livestock Information Sector Analysis and Policy Branch, FAO Agriculture Department Food and Agriculture Organization of the United Nations, Rome; 2002.

3. Hirpa A, Abebe G. Economic significance of sheep and goats. In: Yami, A. and Merkel, R.C, editors. Sheep and goat production handbook for Ethiopia. Ethiopian Sheep and Goat Productivity Improvement Program, Addis Ababa, Ethiopia; 2008.p. 1- 4.

4. Getachew T, Haile A, Tibbo M, Sharma AK, Sölkner J, Wurzinger M. Herd management and breeding practices of sheep owners in a mixed crop-livestock and a pastoral system of Ethiopia. Afr J Agric Res. 2010; 5:685-691.

5. Mohammadabadi MR, Jafari AHD, Bordbar F. Molecular analysis of CIB4 gene and protein in Kermani sheep. Braz J Med Biol Res. 2017; 50: e6177.

6. CSA (Central Statistical Agency). Federal democratic republic of Ethiopia, Agricultural sample survey report on livestock and livestock characteristics, Addis Ababa, Ethiopia; 2020. 188 p.

7. Tibbo M. Productivity and health of indigenous sheep breeds and crossbreds in the Central Ethiopian highlands **[**dissertation on the Internet]**.** Swedish University of Agricultural Sciences; 2006. Available from: https://core.ac.uk/download/pdf/132634398.pdf

8. FAO. The State of the World's Animal Genetic Resources for Food and Agriculture, edited by B.D. Scherf & D. Pilling. The Second Report. FAO Commission on Genetic Resources for Food and Agriculture Assessments, Rome; 2015. Available from : http://www.fao.org/3/a-i4787e/index.html

9. Ibrahim H. Small Ruminant Production Techniques. ILRI Manual 3. International Livestock Research Institute. 1998. Available from : https://books.google.com.et/books?id=RX2lgIq9JooC&pg=PA3&source=gbs_toc_r&cad=3#v=onepage&q&f=false

10. Abebe G. Reproduction in sheep and goats. In: Yami, A. and Merkel, R.C, editors. Sheep and Goat Production Handbook for Ethiopia. Ethiopia Sheep and Goat Productivity Improvement Program, Ethiopia; 2008. pp.60-79.

11. Edea Z. Characterization of Bonga and Horro indigenous sheep breeds of smallholders for designing community based breeding strategies in Ethiopia [master's on the Internet]. Haramaya University; 2008. Available from: https://core.ac.uk/download/pdf/132679007.pdf

12. Kosgey IS, Okeyo AM. Genetic improvement of small ruminants in low-input, smallholder production systems: Technical and infrastructural issues. Small Rumin Res.2007; 70:76-88.

13. IBC (Institute of Biodiversity Conservation). The State of Ethiopia's Farm Animal Genetic Resources. First Country Report. A Contribution to on the State of the World's Animal Genetic Resources, Addis Ababa, Ethiopia; 2004. 7 p.

14. Gizaw S, Komen, Hanotte O, et al. Indigenous sheep resources of Ethiopia: types, production systems and farmers preferences. Anim Genet Resour. 2008; No. 43.

15. Gizaw, S. Sheep breeds of Ethiopia: A guide for identification and utilization. In: Alemu Yami, Kassahun Awgichew, T.A. Gipson and R.C. Merkel, editors. Technical bulletin no.28. Ethiopia Sheep and Goat Productivity Improvement Program, Addis Ababa, Ethiopia; 2009.

16. Gizaw S, Komen H, Oliver H, van Arendonk JAM, Kemp S, Haile A, et al. Characterization and Conservation of Indigenous Sheep Genetic Resources: A Practical Framework for Developing Countries. ILRI. Research Report No. 27. Nairobi, Kenya, ILRI; 2011. Available from : https://cgspace.cgiar.org/handle/10568/5371

17. Taye M, Abebe G, Gizaw S, Lemma S, Mekoya A , Tibbo M, et al. Growth performances of Washera sheep under smallholder management systems in Yilmanadensa and Quarit districts, Ethiopia. Trop Anim Health Prod. 2009; 42: 659–667. https:// DOI 10.1007/s11250-009-9473-x

18. Lauvergne JJ, Bourzat D, Minvielle F. Using morphometric indices to map goat resources. In: The origins and development of African livestock: Archaeology, genetics, linguistics and ethnography, editors. By R M. Blench and K. C. MacDonald). Univ. College London Press, London; 2000. pp. 290-301.

19. Sebsibe A. Sheep and Goat Meat Characteristics and Quality. In: Yami, A. and Merkel, R.C, editors. Sheep and Goat Production Handbook for Ethiopia. Ethiopia Sheep and Goat Productivity Improvement Program, Ethiopia; 2008. pp. 325-340.

20. Worku D, Alemayehu K. On farm and on station comparative performances evaluation of indigenous sheep with their exotic crosses: the case in Dorper and Awassi crosses in Ethiopia. J Appl Anim Sci. 2017; 10(2)

21. Hurni H. Ethiopia Agro-ecological zonation system for selecting soil and water conservation (SWC) options in Ethiopia based on field observations ,Research Report, Agro-ecological Belts of Ethiopia. Centre for Development and Environment University of Bern, Switzerland in association with The Ministry of Agriculture, Ethiopia; 1986. Available from : https://edepot.wur.nl/484855

22. ILCA (International Livestock Centre for Africa). Livestock systems research manual. Working Paper 1, Vol. 1. ILCA, Addis Ababa, Ethiopia; 1990.

23. FAO. Draft guidelines on phenotypic characterization of animal genetic resources. Thirteenth regular session. Commission on genetic resources for food and agriculture, Rome; 2011c.

24. FAO. Phenotypic characterization of animal genetic resources. Guidelines. No. 11. FAO Animal Production and Health, Rome; 2012.

25. Inyangala BAO, Rege JEO, Itulya S. Increasing productivity from indigenous sheep on small scale farms in Kenya. J Zimb Soc Anim Prod. 1992; 4**:** 97-103.

26. Abebe A, Gizaw S, Bisrat A, Goshu S, Besufekad S, Mekonen T, et al. Chanyalew Y. Growth Performance of Dorper and its F1 Crossbreeds. Proceedings of the 8th Annual Regional Conference on Completed Research Activities of Livestock; 2015; Debre Birhan Agricultural Research Center.

27. Wilson RT. Strategies to increase sheep production in east Africa. In: Small Ruminants Production in the Developing Countries. Paper 58. FAO Animal Production and Health, Rome, Italy; 1986c.

28. Kocho T. Production and Marketing Systems of Sheep and Goats in Alaba, Southern Ethiopia [master's on the Internet]. University of Hawassa, Hawassa .2007. Available from : https://core.ac.uk/download/pdf/132631317.pdf

29. SPSS. IBM Corp. Released. IBM SPSS Statistics for Windows, Version 20.0. Armonk, NY: IBM Corp. 2011.

30. Amare T, Goshu G, Tamir B. Lamb Growth Performance Evaluation of Wollo Highland Sheep and Their F1 Crossbreds on Smallholder farm in South Wollo, Ethiopia. Middle East J Sci Res. 2018; 26 (1): 35-45.

31. Abate Z. Performance evaluation of Bonga rams and their progenies in different agro-ecologies of southern Ethiopia [master's on the Internet]. College of agriculture and veterinary medicine, school of graduate students, Jimma University; 2018. Available from : http://repo.mel.cgiar.org:8080/handle/20.500.11766/9481

32. Abegaz S, Gemeda D. Genetic and phenotypic parameters of growth, reproduction, and survival performance of Horro sheep at Bako Agricultural Research Center. Paper presentation; 2000; International Livestock Research Institute.

33. Abegaz S, Awgichew k. Breeds of sheep and goats. In: Yami, A. and Merkel, R.C, editors. Sheep and Goat Production Handbook for Ethiopia. Ethiopia Sheep and Goat Productivity Improvement Program Ethiopia; 2008. pp.10-31.

34. Salifu S. Reproductive performance of djallonké sheep in the northern region of Ghana [master's on the Internet]. kwame nkrumah university of science and technology, Ghana; 2014. Available from : http://ir.knust.edu.gh/bitstream/123456789/6965/1/SADAT%20SALIFU.pdf

35. Bela B, Haile A. Factors affecting growth performance of sheep under village management conditions in the south western part of Ethiopia. Livestock Research for Rural Development. 2009; 21(11): Article #189. .http://www.lrrd.org/lrrd21/11/bela21189.htm

36. Gizaw S, Getachew T, Edea Z, Mirkena T, Duguma G, Tibbo M, et al. Characterization of indigenous breeding strategies of the sheep farming communities of Ethiopia: A basis for designing community-based breeding programs. ICARDA working paper, Aleppo, Syria; 2013. Available from : https://www.researchgate.net/publication/281064132

37. Mukasa-Mugerwa E, Anindo D, Sovani S, Lahlou-Kassi A, Tebely S, Rege JEO , et al. Reproductive performance and productivity of Menz and Horro sheep lambing in the wet and dry seasons in the highlands of Ethiopia. Small Rumin Res. 2002; 4: 261-271.

38. Gebregiorgis A, Guesh Ngash H, Kidane W , Berihu G. Reproductive Performance of Begayt Sheep under Different Management Systems in Western Zone of Tigray. J Dairy Vet Anim Res. 2016; 3(3): 00077. https:// DOI: 10.15406/jdvar.2016.03.00077

Supporting information

S1 Fig. Fig.1 Map of the study districts

S2 Fig. Fig.2 Minimum and maximum values of live weight in kg at different age groups

S1 data. Data of reproductive performance

S2 data. Data of growth performance

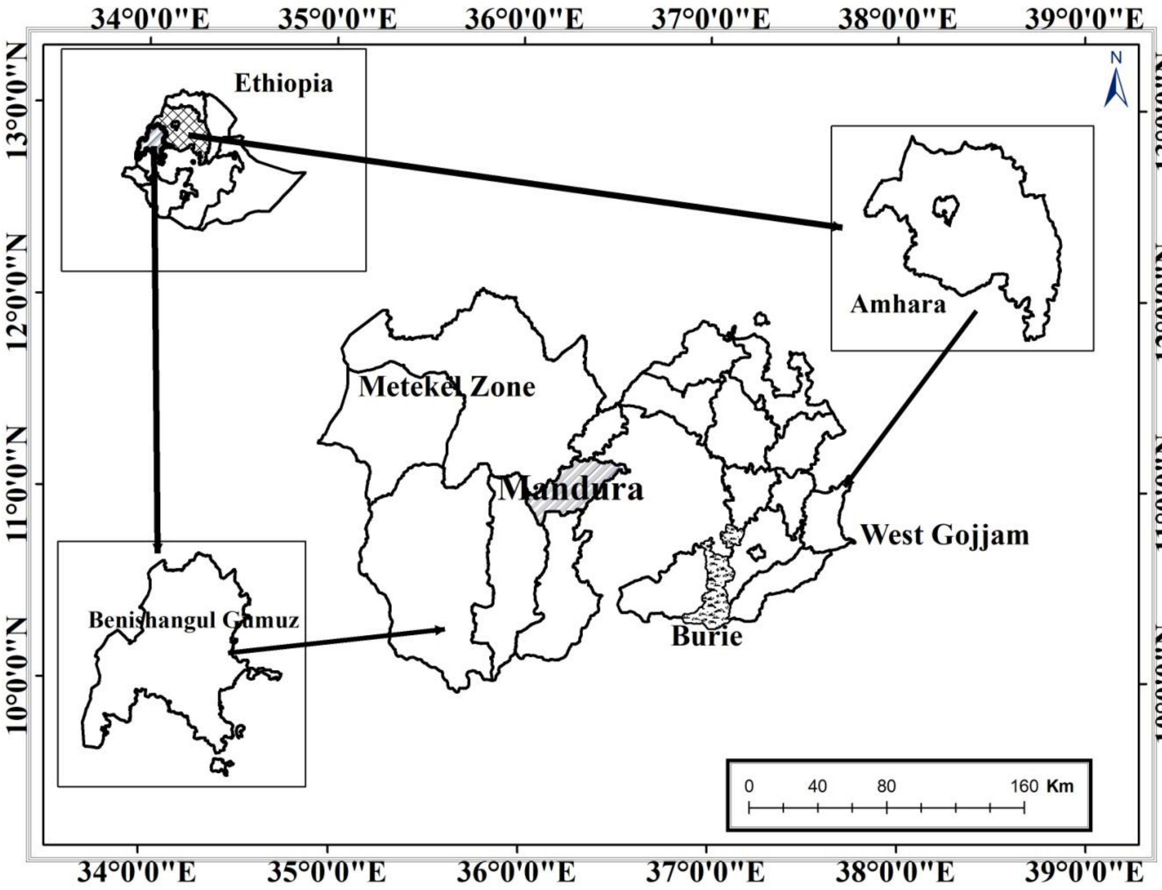

Fig 1. Map of the study districts

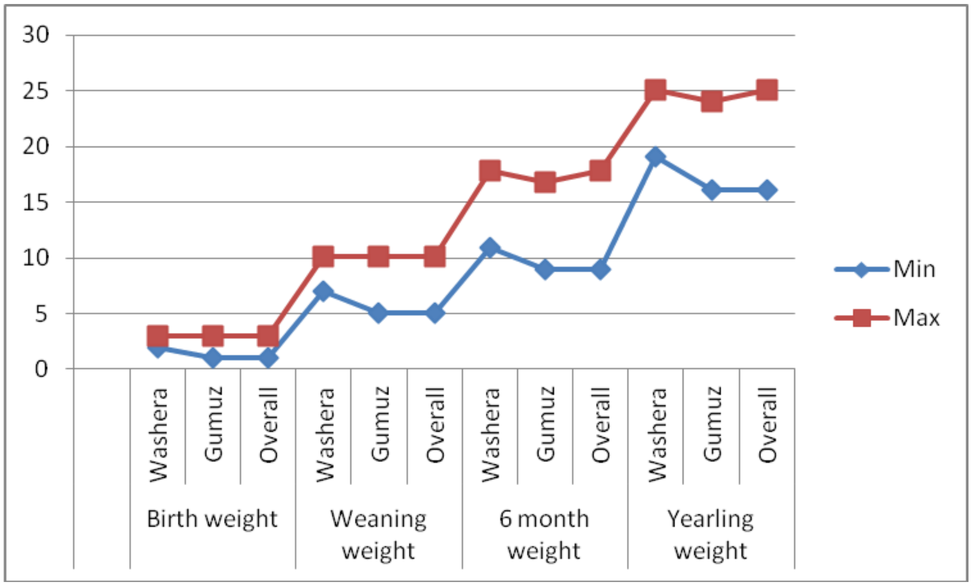

Fig 2. Minimum and maximum values of live weight in kg at different age groups

Click here to access/download
Supporting Information
S1_data. Data of growth performance.xls

Click here to access/download
Supporting Information
S2_data. Data of reproductive performance.xls
