# Supplementary material for: On-farm evaluation of growth and reproductive performances of Washera and Gumuz sheep in northwestern Ethiopia: Basics for setting up breeding objectives/goals
Source: PLoS One. 2021 Jul 20;16(7):e0254924. doi: 10.1371/journal.pone.0254924 (PMC8291677; doi:10.1371/journal.pone.0254924)
Supplement: Attachment — Submitted filename: Response to Reviewers.docx [file pone.0254924.s004.docx]

**Rebuttal letter**

**Response to reviewers**

We thank the editor and reviewers for evaluating our manuscript. We provide line by line responses to comments raised. Please, follow our responses in a yellow mark to every comment/question in green mark. We copied the review decision from submission menu of the editorial manager. We used the same letter to write our responses.

PONE-D-21-11779
On-farm evaluation of growth and reproductive performances of Washera and Gumuz sheep in northwestern Ethiopia: basics for setting up breeding objectives/goals
PLOS ONE

Dear Dr. Asmare,

Thank you for submitting your manuscript to PLOS ONE. After careful consideration, we feel that it has merit but does not fully meet PLOS ONE’s publication criteria as it currently stands. Therefore, we invite you to submit a revised version of the manuscript that addresses the points raised during the review process.

A careful edition of English must be done. Article present potential however must follow a first round of deep revision.

**Response:** Authors express gratitude to the editor for their appreciation

Substantial edition of English grammar has been made including the reviewers comment

Please submit your revised manuscript by Jun 26 2021 11:59PM. If you will need more time than this to complete your revisions, please reply to this message or contact the journal office at plosone@plos.org. When you're ready to submit your revision, log on to https://www.editorialmanager.com/pone/ and select the 'Submissions Needing Revision' folder to locate your manuscript file.

Please include the following items when submitting your revised manuscript:

- A rebuttal letter that responds to each point raised by the academic editor and reviewer(s). You should upload this letter as a separate file labeled 'Response to Reviewers'.
- A marked-up copy of your manuscript that highlights changes made to the original version. You should upload this as a separate file labeled 'Revised Manuscript with Track Changes'.
- An unmarked version of your revised paper without tracked changes. You should upload this as a separate file labeled 'Manuscript'.

If you would like to make changes to your financial disclosure, please include your updated statement in your cover letter. Guidelines for resubmitting your figure files are available below the reviewer comments at the end of this letter.

**Response**

We have included the revised ‘Financial Disclosure Statement’ in our cover letter

If applicable, we recommend that you deposit your laboratory protocols in protocols.io to enhance the reproducibility of your results. Protocols.io assigns your protocol its own identifier (DOI) so that it can be cited independently in the future. For instructions see: <http://journals.plos.org/plosone/s/submission-guidelines#loc-laboratory-protocols>. Additionally, PLOS ONE offers an option for publishing peer-reviewed Lab Protocol articles, which describe protocols hosted on protocols.io. Read more information on sharing protocols at <https://plos.org/protocols?utm_medium=editorial-email&utm_source=authorletters&utm_campaign=protocols>.

We look forward to receiving your revised manuscript.

Kind regards,

Carlos E. Ambrósio, Ph.D
Academic Editor
PLOS ONE


Additional Editor Comments:

A careful edition of English must be done. Article present potential however must follow a first round of deep revision.

**Response:** Authors express gratitude to the editor for their appreciation

Journal Requirements:

When submitting your revision, we need you to address these additional requirements.

1. Please ensure that your manuscript meets PLOS ONE's style requirements, including those for file naming. The PLOS ONE style templates can be found at

https://journals.plos.org/plosone/s/file?id=wjVg/PLOSOne_formatting_sample_main_body.pdf and

https://journals.plos.org/plosone/s/file?id=ba62/PLOSOne_formatting_sample_title_authors_affiliations.pdf

**Response**

The manuscript has been revised to meet all of PLOS ONE’s style requirements.

2. We note that the grant information you provided in the ‘Funding Information’ and ‘Financial Disclosure’ sections do not match.

When you resubmit, please ensure that you provide the correct grant numbers for the awards you received for your study in the ‘Funding Information’ section.

3. We note that Figure 1 in your submission contain map images which may be copyrighted. All PLOS content is published under the Creative Commons Attribution License (CC BY 4.0), which means that the manuscript, images, and Supporting Information files will be freely available online, and any third party is permitted to access, download, copy, distribute, and use these materials in any way, even commercially, with proper attribution. For these reasons, we cannot publish previously copyrighted maps or satellite images created using proprietary data, such as Google software (Google Maps, Street View, and Earth). For more information, see our copyright guidelines: http://journals.plos.org/plosone/s/licenses-and-copyright.

We require you to either (1) present written permission from the copyright holder to publish these figures specifically under the CC BY 4.0 license, or (2) remove the figures from your submission:

**Response**

Figure 1 has been removed from our submission

3.1.    You may seek permission from the original copyright holder of Figure 1 to publish the content specifically under the CC BY 4.0 license.

We recommend that you contact the original copyright holder with the Content Permission Form (http://journals.plos.org/plosone/s/file?id=7c09/content-permission-form.pdf) and the following text:

“I request permission for the open-access journal PLOS ONE to publish XXX under the Creative Commons Attribution License (CCAL) CC BY 4.0 (http://creativecommons.org/licenses/by/4.0/). Please be aware that this license allows unrestricted use and distribution, even commercially, by third parties. Please reply and provide explicit written permission to publish XXX under a CC BY license and complete the attached form.”

Please upload the completed Content Permission Form or other proof of granted permissions as an "Other" file with your submission.

In the figure caption of the copyrighted figure, please include the following text: “Reprinted from [ref] under a CC BY license, with permission from [name of publisher], original copyright [original copyright year].”

3.2.    If you are unable to obtain permission from the original copyright holder to publish these figures under the CC BY 4.0 license or if the copyright holder’s requirements are incompatible with the CC BY 4.0 license, please either i) remove the figure or ii) supply a replacement figure that complies with the CC BY 4.0 license. Please check copyright information on all replacement figures and update the figure caption with source information. If applicable, please specify in the figure caption text when a figure is similar but not identical to the original image and is therefore for illustrative purposes only.

The following resources for replacing copyrighted map figures may be helpful:

USGS National Map Viewer (public domain): http://viewer.nationalmap.gov/viewer/

The Gateway to Astronaut Photography of Earth (public domain): http://eol.jsc.nasa.gov/sseop/clickmap/

Maps at the CIA (public domain): https://www.cia.gov/library/publications/the-world-factbook/index.html and https://www.cia.gov/library/publications/cia-maps-publications/index.html

NASA Earth Observatory (public domain): http://earthobservatory.nasa.gov/

Landsat: http://landsat.visibleearth.nasa.gov/

USGS EROS (Earth Resources Observatory and Science (EROS) Center) (public domain): http://eros.usgs.gov/#

Natural Earth (public domain): http://www.naturalearthdata.com/

[Note: HTML markup is below. Please do not edit.]

Reviewers' comments:

Reviewer's Responses to Questions

**Comments to the Author**

1. Is the manuscript technically sound, and do the data support the conclusions?

The manuscript must describe a technically sound piece of scientific research with data that supports the conclusions. Experiments must have been conducted rigorously, with appropriate controls, replication, and sample sizes. The conclusions must be drawn appropriately based on the data presented.

Reviewer #1: Yes

Reviewer #2: Yes

2. Has the statistical analysis been performed appropriately and rigorously?

Reviewer #1: Yes

Reviewer #2: Yes

3. Have the authors made all data underlying the findings in their manuscript fully available?

The [PLOS Data policy](http://www.plosone.org/static/policies.action#sharing) requires authors to make all data underlying the findings described in their manuscript fully available without restriction, with rare exception (please refer to the Data Availability Statement in the manuscript PDF file). The data should be provided as part of the manuscript or its supporting information, or deposited to a public repository. For example, in addition to summary statistics, the data points behind means, medians and variance measures should be available. If there are restrictions on publicly sharing data—e.g. participant privacy or use of data from a third party—those must be specified.

Reviewer #1: Yes

Reviewer #2: Yes

4. Is the manuscript presented in an intelligible fashion and written in standard English?

PLOS ONE does not copyedit accepted manuscripts, so the language in submitted articles must be clear, correct, and unambiguous. Any typographical or grammatical errors should be corrected at revision, so please note any specific errors here.

Reviewer #1: Yes

Reviewer #2: Yes

5. Review Comments to the Author

Please use the space provided to explain your answers to the questions above. You may also include additional comments for the author, including concerns about dual publication, research ethics, or publication ethics. (Please upload your review as an attachment if it exceeds 20,000 characters)

Reviewer #1: Please reviewed manuscript attached file including comments. Moreover, it is better to edit manuscript by a native English person شnd in terms of point, comma, distance, etc., it is better to be carefully corrected and reviewed.

**Response**

Authors express gratitude to the reviewer for their comments.

We have tried to incorporate all the comments line by line. We look forward to learn more from you. Substantial edition of English grammar has been made including the reviewers comment ( commas, space, distance etc) (Please see the revised manuscript)

Reviewer #2: General comments:

The manuscript shows the evaluation of performance and reproductive traits in farm conditions for Washera and Gumuz sheep. The results demonstrate the variation between the two breeds evaluated and the sex of the animals, and this interesting information can be used to guide possible breeding programs for sheep in Ethiopia.
The writing format of the text needs revision, adapting to scientific norms.

**Response**

Authors express gratitude to the reviewer for their comments.

Substantial changes have been made by revising writing format of the text and describing results in tabular form. For instance, table 2 was included to describe the reproductive performance results (see the revised manuscript).

Specific comments:

Abstract
The division of topics in the abstract is very disproportionate, with the results occupying more than 50% of the text. The conclusion has to be less. The mean values and standard deviation can be excluded and shown only the factors that affected performance and reproductive characteristics. Therefore, it would be important to rewrite lines 28 to 37.

L37-39: Please, delete.

**Response**

Authors express gratitude to the reviewer for their best comment. We have tried to keep the proportionality between result, methodology and conclusion taking the respected reviewer comment into account. In this regard, we have excluded mean values and standard deviation, we have reduced the text from the result and conclusion part and we tried to re-write lines 28 to 37.

Lines 37 to 39 were deleted from abstract (page-2)

Introduction
The introduction is well written and the main important points for the work have been made. However, it is possible to reduce the text of the introduction by making it more concise.
L48: TLU? Define
L89: on - farm. L91 on farm. L92 on-farm, please use only one.
L97-101: The hypotheses are not well supported by the introduction. I think you can delete this part of the text.

**Response**

Authors express gratitude to the reviewer for their appreciation. We have tried to condense the introduction by reducing some texts including the hypothesis.

L48: TLU has been defined into “tropical livestock unit” (page-2, line 52)

L91 “on farm” has been corrected in to “on – farm” (page -4, lines 93, 95, and 96)

L97-101: We have deleted the hypotheses from the introduction (page-4)

Material and Methods
L163-169: Please, delete
L198-201: Please, delete
L209-210: Please, delete

**Response**

L163-169- we have deleted (page-6)

L198-201- we have deleted (page-6)

L209-210 - we have deleted (page-6)

Results/Discussion
The results and discussion of the present work should be brought together in a single topic. The performance results are presented in table form, so there is no need to describe them in the text. Figure 2 was confused, I believe it can be deleted. Table 1 must not be in the proper format of PlosONE, so the form of visualization must be adjusted and improved.
The presentation of reproductive characteristics should also be in a table format for better visualization, including the probability values, just as it was done with the performance results.

**Response**

Results / Discussion part has been brought together into a single topic “Results and Discussion” (page-8, line 227).Texts in growth performance results have been reduced except texts in growth performance results not described in tabular form. These are results regarding pre- weaning average daily weight gain and post weaning average daily weight gain (page-8, lines 234-239). We have deleted Figure 2. Visualization of table1 has been adjusted and improved by increasing row height (page-10, lines 268-292). The presentation of reproductive characteristics has been summarized into a table format for better visualization, including the probability values (Table 2, page-11, lines 300-305).

The discussion is good.
L328-340: This part should be in material and methods, please include in the correct topic.

**Response**

Authors express gratitude to the reviewer for their appreciation. L328-340 has been moved to material and methods by including the corrected topic (page-6, line 175)

Conclusion
The conclusion is repeating what has already been put in the results.
L447-455: Please, delete
L459-461: Please, delete

**Response**

We have deleted L447-455 from the conclusion (page-14)

We have deleted L459-461 from the conclusion (page-14)

6. PLOS authors have the option to publish the peer review history of their article ([what does this mean?](https://journals.plos.org/plosone/s/editorial-and-peer-review-process#loc-peer-review-history)). If published, this will include your full peer review and any attached files.


If you choose “no”, your identity will remain anonymous but your review may still be made public.


**Do you want your identity to be public for this peer review?** For information about this choice, including consent withdrawal, please see our [Privacy Policy](https://www.plos.org/privacy-policy).

Reviewer #1: No

Reviewer #2: No

[NOTE: If reviewer comments were submitted as an attachment file, they will be attached to this email and accessible via the submission site. Please log into your account, locate the manuscript record, and check for the action link "View Attachments". If this link does not appear, there are no attachment files.]

While revising your submission, please upload your figure files to the Preflight Analysis and Conversion Engine (PACE) digital diagnostic tool, <https://pacev2.apexcovantage.com/>. PACE helps ensure that figures meet PLOS requirements. To use PACE, you must first register as a user. Registration is free. Then, login and navigate to the UPLOAD tab, where you will find detailed instructions on how to use the tool. If you encounter any issues or have any questions when using PACE, please email PLOS at [figures@plos.org](mailto:figures@plos.org). Please note that Supporting Information files do not need this step.

*In compliance with data protection regulations, you may request that we remove your personal registration details at any time.* [*(Remove my information/details)*](https://www.editorialmanager.com/pone/login.asp?a=r)*. Please contact the publication office if you have any questions.*
